# Magneto-hydrodynamics of multi-phase flows in heterogeneous systems with large property gradients

**DOI:** 10.1038/s41598-021-97177-8

**Published:** 2021-09-23

**Authors:** T. F. Flint, M. C. Smith, P. Shanthraj

**Affiliations:** 1 The Dalton Nuclear Institute, The University of Manchester, Manchester, UK; 2 The Department of Materials, The University of Manchester, Manchester, UK

**Keywords:** Fluid dynamics, Nuclear astrophysics, Astrophysical plasmas, Magnetically confined plasmas, Magnetospheric physics, Phase transitions and critical phenomena, Thermodynamics, Nuclear fusion and fission, Permeation and transport

## Abstract

The complex interplay between thermal, hydrodynamic, and electromagnetic, forces governs the evolution of multi-phase systems in high technology applications, such as advanced manufacturing and fusion power plant operation. In this work, a new formulation of the time dependent magnetic induction equation is fully coupled to a set of conservation laws for multi-phase fluid flow, energy transport and chemical species transport that describes melting and solidification state transitions. A finite-volume discretisation of the resulting system of equations is performed, where a novel projection method is formulated to ensure that the magnetic field remains divergence free. The proposed framework is validated by accurately replicating a Hartmann flow profile. Further validation is performed through correctly predicting the experimentally observed trajectory of Argon bubbles rising in a liquid metal under varying applied magnetic fields. Finally, the applicability of the framework to technologically relevant processes is illustrated through the simulation of an electrical arc welding process between dissimilar metals. The proposed framework addresses an urgent need for numerical methods to understand the evolution of multi-phase systems with large electromagnetic property contrast.

## Introduction

Magneto-hydrodynamics (MHD) describes important physical phenomena spanning many length scales from biological systems to astrophysical phenomena such as stellar interiors, solar flares, and planetary magnetic field generation^1–4^. MHD also describes technologically important applications such as the magnetic confinement of fusion plasma, the interaction of fusion plasma’s with proposed liquid metal blankets, and lower temperature applications such as electric arc welding and joining processes^5–8^. The governing equations in MHD describe the conservation of mass, momentum and energy transfer, and the transport of the magnetic field intensity through augmentation with the low-frequency approximation of Maxwell’s equations^9–11^. These systems are strongly coupled, highly nonlinear and characterised by coupled physical phenomena that span a very large range of length and timescales. These characteristics make the scalable, robust, accurate, and efficient computational solution of these systems extremely challenging^9,11,12^. The influence of magnetic fields on free-surface liquid metal flows is extremely important in advanced manufacturing; these processes are governed by a combination of electro-magnetic, thermal and fluid-dynamic driving forces.

In metal additive manufacturing, or electric arc welding, an electrical arc is generated through a partially insulating gas phase between an electrode and the substrate material^13,14^. The arc discharge causes a rapid increase in temperature through the plasma column and substrate; causing melting, topological changes due to fluid motion, mixing in the case where dissimilar metallic phases are present, and the generation of highly complex flow patterns due to the interplay between the induced magnetic field, temperature field and velocity field^15^. Once the electrical arc is extinguished, the portion of the metallic substrate that melted will begin to solidify and any changes caused by the melting events will be inherited by the substrate^14,16^. Mathematically describing this metal additive manufacturing scenario, or any scenario with spatial gradients in electromagnetic properties requires a formulation of the conservation law for magnetic field strength that captures these gradients properly; it is worth highlighting that large property gradients usually pose serious computational challenges. Such a magnetic induction equation could be used to supplement the conservation of mass, momentum and energy transfer equations that describe the evolution of a system containing a mixture of chemical components. With such a mathematical framework it would be possible to accurately predict the evolution of material systems subjected to hydrodynamic and electromagnetic driving forces. Such a complete framework describing these systems is absent from the literature as there is no description of the magnetic induction equation, derived to capture scenarios where large spatial gradients in electromagnetic properties exist^9,11,12,17–21^.

In the vast majority of MHD research in the literature, single phase flow is considered, where naturally there are no gradients in electrical conductivity or magnetic permeability in the system; in these cases it is possible to simplify the magnetic induction equation^7,22^. When gradients in material properties are considered, and the full form of the magnetic induction equation is used, it is possible to mathematically capture the effect of these property gradients when they vary smoothly^9^. Where the time variation of the magnetic field is negligible, it is again possible to simplify the induction equation, here the frameworks used consider the electric potential as the main electromagnetic variable^10,23–28^; limiting the applicability of such frameworks to steady processes. For cases where the magnetic field is known to oscillate, such as alternating current joining processes^11,13,15,29^, and oscillations of the liquid metal coolant in fusion reactors, a time dependent and complete form of the induction equation should be used^8,19^.

Electromagnetic phenomena are most commonly formulated and solved using the finite element method (FEM)^12,17,20^; although many finite volume method (FVM) formulations and solution schemes are now present in the literature^18,30,31^; including popular implementations in commercial packages such as Ansys-Fluent^32–34^. FVM is often the favoured solution approach for conservation laws, although obtaining high order schemes is more cumbersome than in the FEM^18^.

In this work, we formulate a MHD framework derived from the continuum Maxwell’s equations for the description of multi-phase fluid mixtures under the influence of magnetic fields. We consider the gradients in electromagnetic properties during the derivation of the magnetic induction equation to produce a general framework capable of describing multi-phase magneto-thermal-hydrodynamic systems. This approach not only permits the simulation of cases with electromagnetic property contrast, but also scenarios where the applied magnetic field varies in time. The projection method is utilised to ensure that the derived magnetic induction equation remains solenoidal within machine accuracy at all times. A multi-phase volume of fluid approach is utilised to describe the evolution of a heterogeneous mixture of chemical species, both metallic and atmospheric, in the computational domain. Melting and fusion state changes are captured within the framework. This paper is organised as follows: in “Results”, representative examples are used to benchmark the proposed method with known analytical and experimental solutions, as well as to showcase its applicability. Representative boundary conditions were applied on the computational domains, which include fluid and solid phases. The field equations are solved over the entire domain, so no special treatment of the fluid-solid boundary is required. The simulation results are discussed, and the conclusions are summarised in “ Discussion”. Finally the MHD model formulation for state transitions in multi-phase mixture systems is presented in “Methods”, followed by an outline of its numerical implementation in “Numerical implementation”. Additional details of the implementation can be found in the Appendix.

## Results

A multi-phase description of the conservation of magnetic field strength is developed and supplemented with a description of the thermal-fluid dynamics for multi-phase mixtures. In this section, the developed framework is first validated against a well known analytical solution for single phase flow subjected to a transverse magnetic field. For the case of multi-phase flows with externally applied magnetic fields, experimentally measured Ar bubble trajectories, in a Ga–In–Sn liquid metal, are used to further validate the framework. Finally the developed framework is applied to the technologically relevant scenario of the electric arc welding of two dissimilar ternary Ni–Al–Fe alloys under an Ar atmosphere. The thermo-physical properties used in the validation and simulation contained within the remainder of this work are shown in Table 1.

**Table 1:** Thermo-physical properties used in the thermal-fluid dynamics simulations^35–39^.

	Ni	Fe	Al	Ga–In–Sn	Ar
$\rho \,\left[ kg\,m^{-3}\right]$	8908	7874	2710	6360	1.2
$\nu \,\left[ m^{2}\,s^{-1}\right]$	$1\times 10^{-7}$	$2.5\times 10^{-7}$	$2\times 10^{-7}$	$3.4\times 10^{-7}$	$1.4\times 10^{-5}$
$k_{solid}\,\left[ kg\,m\,s^{-3}\,K^{-1}\right]$	90.9	80.4	237	−	–
$k_{fluid}\,\left[ kg\,m\,s^{-3}\,K^{-1}\right]$	100	30	150	–	$1.7\times 10^{-2}$
$c_{p_{solid}}\,\left[ m^{2}\,s^{-2}\,K^{-1}\right]$	440	450	897	−	–
$c_{p_{fluid}}\,\left[ m^{2}\,s^{-2}\,K^{-1}\right]$	730	820	1180	–	530
$T_{m}\,\left[ K\right]$	1728	1809	932	−	–
$\beta \,\left[ K^{-1}\right]$	$6.6\times 10^{-5}$	$1.54\times 10^{-5}$	$5\times 10^{-5}$	$1\times 10^{-5}$	$1\times 10^{-4}$
$\sigma _{E}\,\left[ kg^{-1}\,m^{-3}\,s^{3}\,A^{2}\right]$	$1.43\times 10^{7}$	$1.00\times 10^{7}$	$3.77\times 10^{7}$	$3.46\times 10^{6}$	1.0
$\mu _{M}\,\left[ kg\,m\,s^{-2}\,A^{-2}\right]$	$1.26\times 10^{-4}$	$6.3\times 10^{-3}$	$1.256\times 10^{-6}$	$8.0\times 10^{-7}$	$1.256\times 10^{-6}$

### Single phase hartmann flow

**Figure 1:**
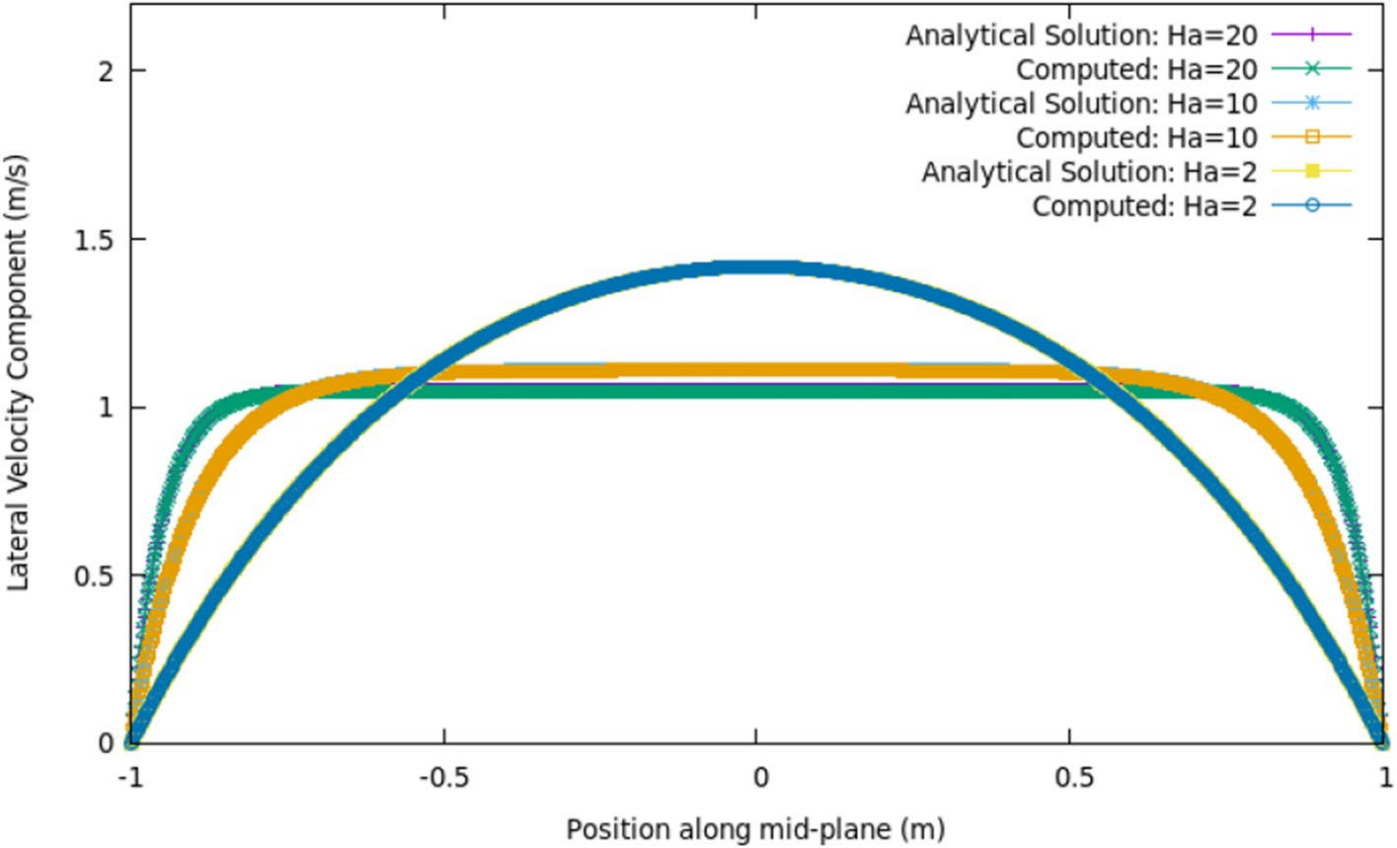
Analytical solution and numerically computed solution to Hartmann flow problem, at $t=2$ s.

The most appropriate method by which to validate any numerical framework is an analytical solution. Therefore a well known MHD problem of single phase flow in the presence of a magnetic field, for which an analytical solution exists, was investigated in the first instance. In this scenario, an electrically conducting fluid flows between two parallel plates, with a magnetic field applied perpendicular to the flow direction. This scenario is known as the Hartmann flow, and is well documented, as is the analytical solution to the flow profile and often used as a benchmark solution for validation of MHD codes^40^. For brevity, in this case the density, viscosity, electrical conductivity and magnetic permeability are all set to unity, such that the characteristic Hartmann number (Ha) becomes equal to the magnitude of the applied magnetic field. A two dimensional domain was considered; the parallel plates were separated by a distance of 2 m, and a flow length of 20 m was simulated. 200 cells were present in the flow direction, and 80 cells bridged the distance between the plates for a total of 16,000 cells in the computational domain. Figure 1 shows the comparison between the analytical solution^40^, and the numerically computed flow profile along a line at the mid-length of the domain, normal to the flow direction.

As the strength of the applied magnetic field is increased from 2 to 20 T, the magnitude of the Lorentz force increases, opposing the flow and reducing the peak velocity magnitude from 1.42 to 1.05 m s$^{-1}$ . At $Ha=20$, the flow profile is almost constant through the channel (except very close to the walls so the solution satisfies the no slip boundary condition). It can be seen from Fig. 1 that the framework is in strong agreement with the analytical solution for a range of applied magnetic field strengths.

### Bubble rise in a magnetic field

For multi-phase mixture MHD flows analytical solutions are not available. Therefore, to further validate the framework for scenarios where property gradients exist, the scenario of Ar gas bubbles, rising in Ga–In–Sn liquid metal with increasing applied magnetic field strength are investigated. Many experimental studied on gaseous bubble rise in magnetic fields have been performed^41–43^. We have chosen to validate against the work of Richter et al, which is fully described elsewhere^43^, due to the larger number of experimental cases, and greater magnitude of applied magnetic field. In summary, a transverse magnetic field is applied, normal to the direction of gravity, through a liquid column of Ga–In–Sn alloy. Ar bubbles are introduced at the base of the column, and rise through the liquid metal. The positions of the bubbles as they rise through the alloy were measured by an ultrasound transit time technique and X-ray radiography. The dimensions of the experimental vessel were 144 mm deep, 144 mm wide, and 12 mm thick; with bubbles of diameter $\sim \,5.2$ mm introduced at the base of the domain. Given that the thickness of the domain was 12 mm, presumably for better signal detection in the experiment, the motion of the bubbles as they rise through the column was found by Richter et al. to be planar, indeed in the authors conclusions they remark “The bubbles performed a planar zigzag motion with a lateral drift during the rise”. For this reason, in this work the computational domain has been simplified to a two dimensional representation of the experimental set up. In the future, more complex validation cases will be investigated in three dimensions, where appropriate.

**Figure 2:**
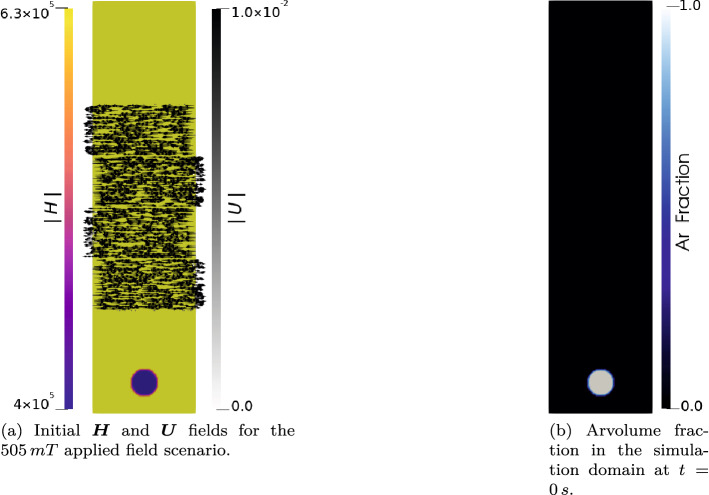
Initial Ar fraction and velocity perturbation used in the bubble rise simulations. The initial ${\varvec{H}}$ field is also shown for the 505 mT case. In all four cases the initial ${\varvec{H}}$ field is set such that ${\varvec{B}}$ is uniform at $t=0$ s and equal to the magnitude of the applied field.

A computational domain of 80 mm in depth, and 20 mm wide, is discretised into 25,600 computational cells to simulate this process. In the simulations a non-uniform magnetic field strength ${\varvec{H}}$ was initialised at $t=0$ s, in such a manner as to create a uniform magnetic field ${\varvec{B}}$ at $t=0$ s equal to the applied ${\varvec{B}}$ experimentally. The fields in the domain then evolve over time. Four of the cases reported by Richter et al. are simulated, namely the cases with 0 mT, 99 mT, 242 mT and 505 mT applied magnetic field. In order to capture the low magnitude velocity field perturbations that would have been present in the experimental liquid column, due to the passage of the previous bubbles in Richter’s experiment, at $t=0$ s small transverse velocity perturbations are initiated in the domain; this removes the requirement for simulation of the entire experimental domain. The same initial velocity perturbation is applied in all cases. Figure 2a,b show the initial velocity profile, and Ar volume fraction for all cases, as well as the initial ${\varvec{H}}$ field for the 505 mT case.

**Figure 3:**
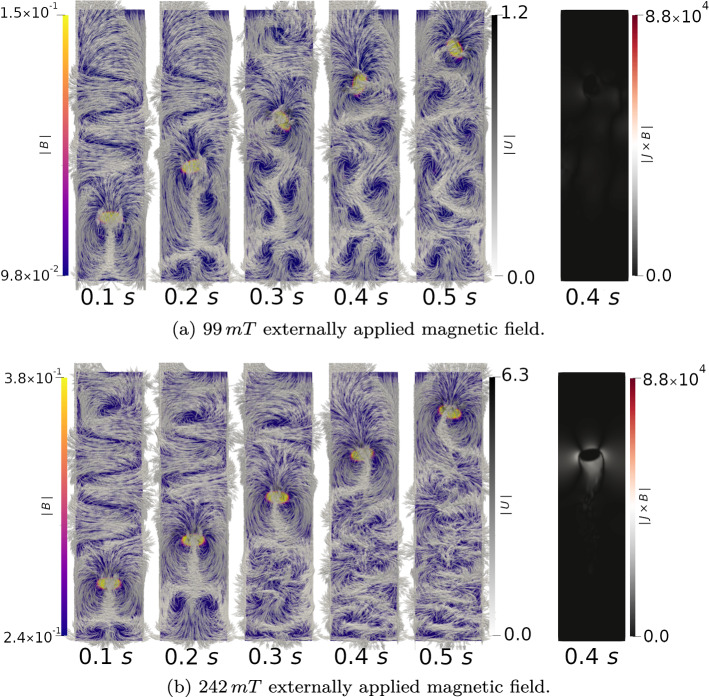
Simulated single Ar bubble rise through Ga–In–Sn liquid with 99 mT and 242 mT applied magnetic field with $\left| {\varvec{B}}\right|$ and ${\varvec{U}}$ vectors. The simulated behaviour of the system strongly agrees with that observed experimentally. With no applied magnetic field, the bubble rises and instabilities develop causing oscillations. As the magnitude of the applied horizontal magnetic field is increased, the Lorentz force, ${\varvec{J}}\times {\varvec{B}}$, dampens out the magnitude of these instabilities to a greater degree, until at 505 mT the bubble rises through the liquid metal almost perfectly linearly. Animations of these simulations accompany this work, and show this oscillation very clearly.

The evolution of the Ga–In–Sn–Ar system as a function of time is shown in Fig. 3 for the 99 mT and 242 mT cases. Also shown in Fig. 3 are the magnitudes of the Lorentz force, ${\varvec{J}}\times {\varvec{B}}$, at 0.4 s.

As can be seen in Fig. 3, the Ar bubble rises through the Ga–In–Sn due to the density difference between the bubble and the alloy. The surface tension force, acting at the interface of the bubble and the liquid metal, acts to reduce the bubble surface area and prevent break-up during the rise. In the 99 mT case, the tumbling behaviour of the bubble is much more pronounced, as was documented in the experimental observations. The vorticity in the wake of the bubble decreases as the applied field intensity is increased; it can be seen that the velocity field is significantly dampened in the liquid metal, due to the increase in the magnitude of the Lorentz force, from 1.2 m s$^{-1}$ in the 99 mT case to 0.63 m s$^{-1}$ in the 242 mT case. The magnitude of the Lorentz force is observed to increase from $6.9\times 10^{3}$ N m$^{-3}$ at 99 mT to $9.9\times 10^{4}$ N m$^{-3}$ at 505 mT. The Lorentz force opposes the buoyancy of the Ar bubble, reducing its effective vertical rise velocity to 0.124 m s$^{-1}$, 0.123 m s$^{-1}$ and 0.088 m s$^{-1}$ for applied fields of 99 mT, 242 mT and 505 mT, respectively. In addition to reducing the effective velocity, the Lorentz force also stabilises lateral oscillations with the oscillation amplitude reducing from 9 mm at 0 mT to 7 mm at 99 mT and 1.7 mm at 242 mT. At 505 mT, no lateral oscillations were observed. The computed bubble behaviours, using the presented framework are in very good agreement with those experimentally measured.

**Figure 4:**
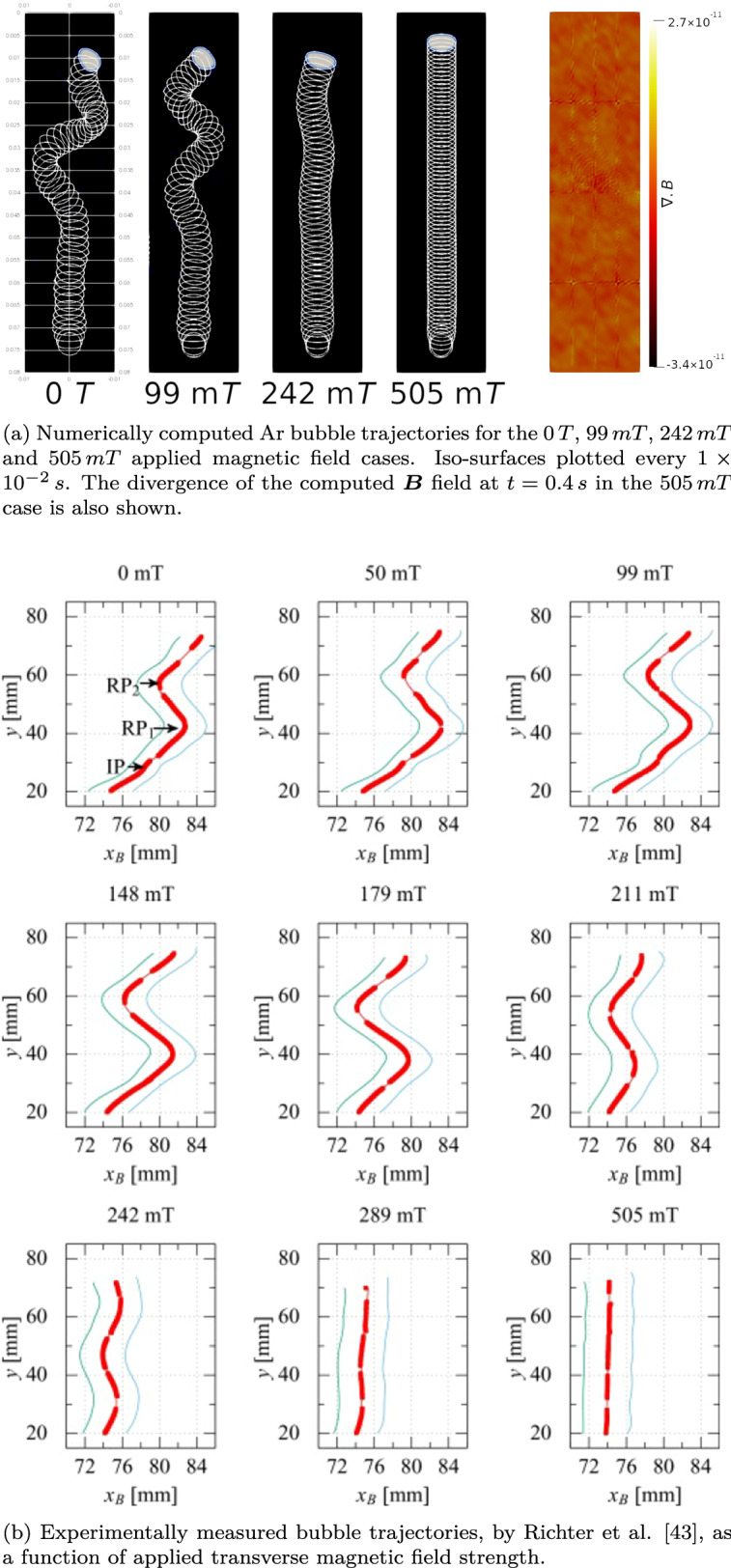
Comparison between numerically computed, and experimentally measured Ar bubble trajectories in Ga–In–Sn liquid metal, for various applied magnetic field strengths.

Figure 4 shows the comparison between the numerically computed, and experimentally measured bubble trajectories. Also shown is the solenoidal property of the numerically computed magnetic field, demonstrating the the magnetic conservation equation is upheld by the proposed framework. This prevents the generation of spurious magnetic currents affecting the flow field and deviating the simulation from an accurate description of the system^44^. As can be seen, $\nabla \cdot {\varvec{B}}$ in the domain is maintained at negligible levels, comparable with those reported in similar work^9^.

As can be seen in Fig. 4, as the magnitude of the applied magnetic field is increased, the lateral oscillation magnitude of the rising bubble decreases, due to the increasing magnitude of the Lorentz force.

### Electrical arc welding

**Figure 5:**
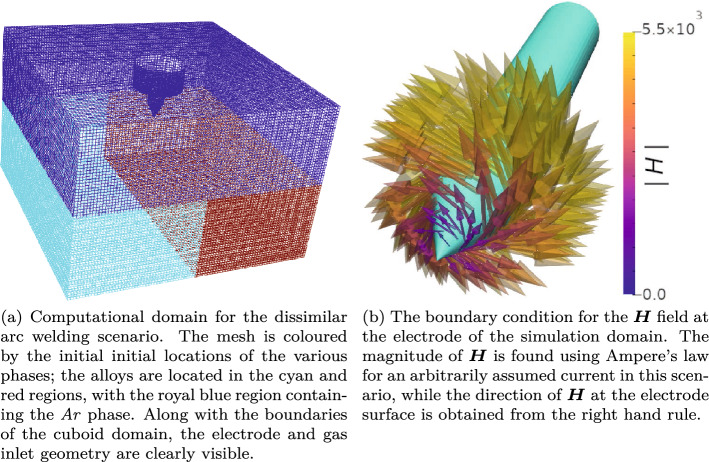
Computational domain for the electric arc welding case (**a**), showing an exploded view of the electrode and the boundary condition in ${\varvec{H}}$ applied at the electrode surface (**b**).

One of the most exciting areas where the presented framework may be used to predict system evolution, is in advanced manufacturing scenarios, where high energy density electrical sources are used to induce state change in metallic substrates. Although arc welding of metallic components has been performed for over a century, the complex velocity, momentum and electromagnetic fields involved means that the process is still poorly understood from a physical perspective. This has meant that the development of welding technologies has largely been based on experimental trial and error. In this section, we use the derived framework to predict the behaviour in a dissimilar metal arc weld. Here, two alloys are present in the domain under an inert argon atmosphere. The alloys investigated both contain Fe, Al and Ni with differing compositions. Alloy 1 is composed of $30\%$ Ni, $65\%$ Al and $5\%$ Fe; alloy 2 is composed of $90\%$ Ni, $8\%$ Al and $2\%$ Fe. The computational domain, highlighting the initial regions of the Ar region and alloy regions, is shown in Fig. 5a. An exploded view of the electrode, and the boundary condition in ${\varvec{H}}$ at the electrode surface are also shown in Fig. 5b. The domain is 20 mm deep, 30 mm wide, and 30 mm long. The domain is decomposed into 490,057 computational cells. In the electrical arc simulation, the domain was decomposed into 512 processors.

**Figure 6:**
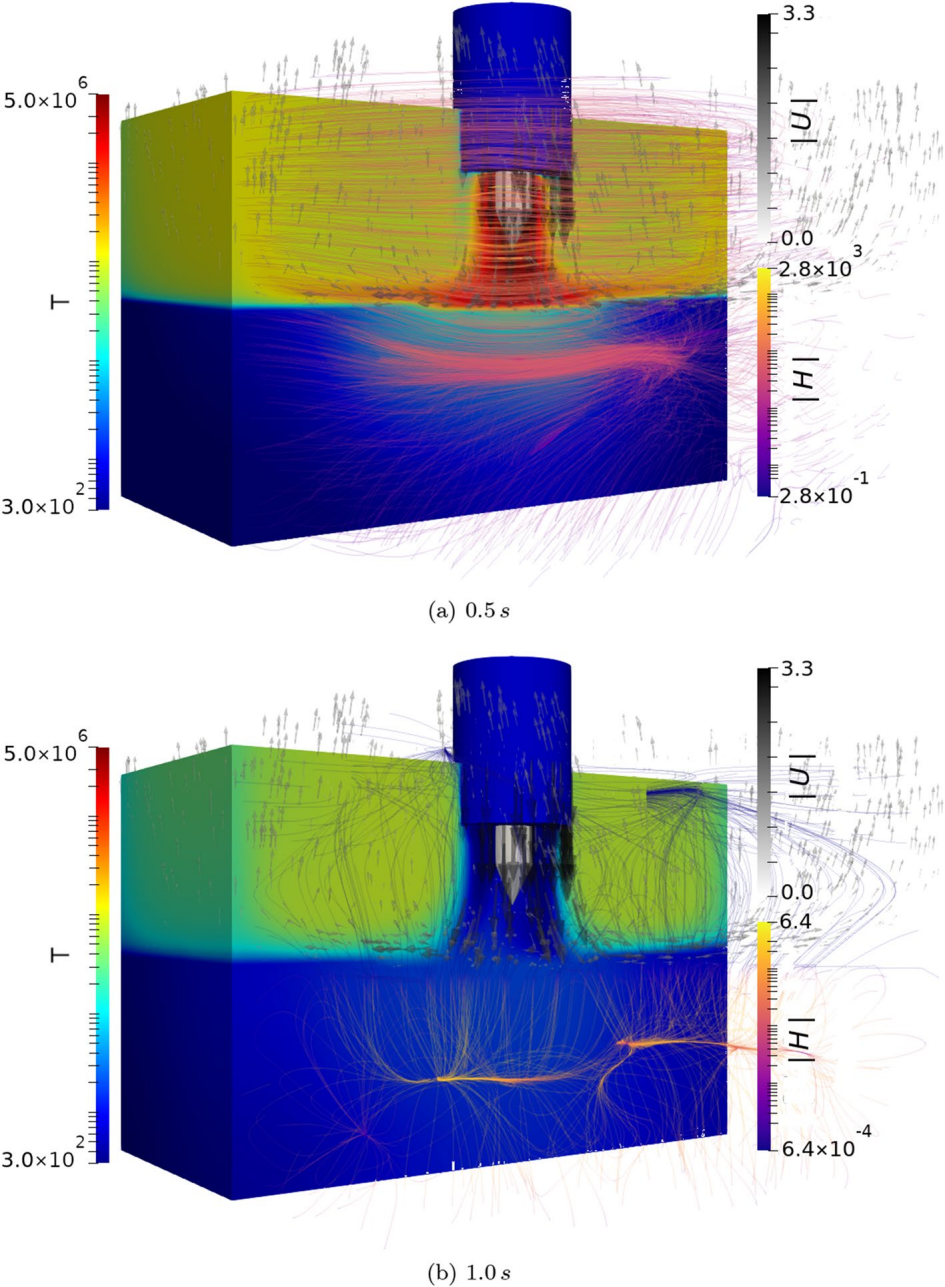
Evolution of the ${\varvec{U}}$, ${\varvec{H}}$ and *T* fields inside the computational domain. The magnitude of the *H* field, initially set to $5.3\times 10^{3}$ A m$^{-1}$ is set to 0 A m$^{-1}$ at $t=$ 0.52 s. As the current density rapidly decreases after this time, so does the Ohmic heating effect, and therefore the temperature in the vicinity of the weld-pool decreases. Here ${\varvec{U}}$ glyphs are plotted, along with stream lines for the ${\varvec{H}}$ field.

The boundary condition for ${\varvec{H}}$ at the electrode surface, is found using Ampere’s law, for a known current. In this work a current of 33.5 A is arbitrarily assumed to minimise heat input and allow a smaller computational domain to be used, therefore the magnitude of ${\varvec{H}}$ at the electrode surface is $5.3\times 10^{3}$ A m$^{-1}$ as the electrode has a maximum radius of $1\,mm$. This is applied as a vortical boundary condition at the electrode surface, as shown in Fig. 5b. This highly vortical ${\varvec{H}}$ field at the electrode surface induces a large current density, $\left( {\varvec{J}}=\nabla \times {\varvec{H}}\right)$ in the vicinity of the electrode. This large current density is the primary mechanism of heat generation in the simulation domain, through the Ohmic heating term, ${\varvec{J}}\cdot {\varvec{J}}/\sigma _{E}$, in Eq. (3). In this arc welding scenario, the temperature of the inlet gas is assumed to be 300 K, with an inlet velocity of $3\,m\,s^{-1}$ through the gas-cup (shown in Fig. 5a), which are applied as boundary conditions.Additionally the boundary conditions on the bottom wall and four bounding walls for ${\varvec{U}}$ were assumed to be slip conditions (note that the metallic substrate did not melt to the domain boundary and the momentum damping term dominates the momentum equation in solidified substrate). Furthermore, the boundary conditions for ${\varvec{H}}$ applied at the remaining domain boundaries were of the Neumann type. A thermal extraction boundary condition was applied at the bottom domain boundary with a value of $dT/dX = -1\times 10^3$ K m$^{-1}$ to simulate thermal conduction out of the domain. The evolution of the ${\varvec{H}}$, ${\varvec{U}}$ and *T* fields inside the computational domain are shown in Fig. 6. At $t=0.52$ s the magnitude of ${\varvec{H}}$ at the electrode surface was reduced to 0 A m$^{-1}$, in order to capture the cooling and solidification behaviour of the metallic substrate.

**Figure 7:**
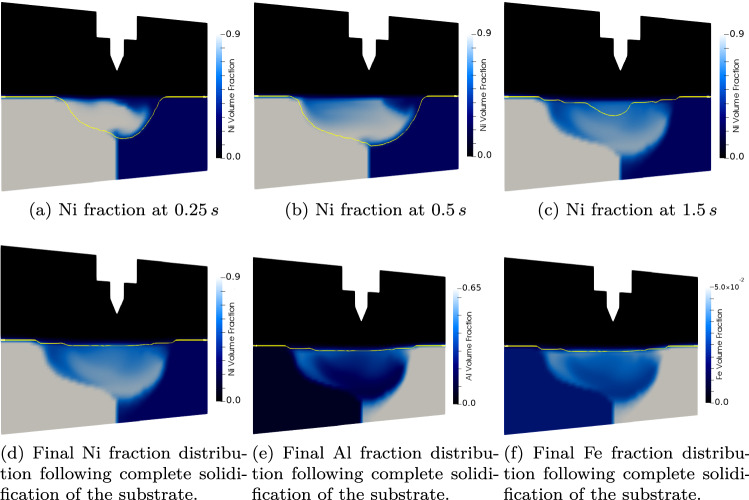
Temporal evolution of the phase fractions at a cross section through the mid plane of the domain, normal to the initial boundary between alloys 1 and 2. The boundary between the solid and fluid regions is shown in yellow. As more of the substrate melts, and the convective flow is established in the metal, the two alloys begin to mix together, prior to solidification. Note that the difference in melting temperatures is evident from the melt-pool shape.

As can be seen from Fig. 6; the magnetic field propagates from the electrode, through the Ar phase and, after some time, into the lower magnetic diffusivity metallic substrate. The heat generation at the electrode surface is transported towards the metallic substrate. This heating causes the temperature to rise rapidly in the vicinity of the electrode and causes localised melting and fluid flow between the two alloys. The alloy with the higher Al content begins to melt at a lower temperature, and experiences a greater degree of melting. As the two alloys melt, a combination of surface tension, incident gas velocity at the interface, and buoyancy forces generate velocity fields within the molten substrate that aid in the mixing of the two alloys; in conjunction with the diffusive mixing. Following the extinction of the arc (when ${\varvec{H}}$ falls to zero at the electrode surface), the current density in the vicinity of the electrode rapidly falls. As the Ohmic heating effect vanishes, the molten metallic substrate begins to solidify, as heat is lost to the solid regions, and to the domain boundaries. Following the arc extinction, the magnetic field in the domain rapidly diffuses away; the magnetic field induced in the metallic substrate takes longer to dissipate, as can be seen in Fig. 6, as the magnetic diffusivity is lower in the metallic regions. The evolution of the Ni volume fraction is shown at a cross section of the computational domain, for various time-steps in Fig. 7a–c. As the alloys flow together and mix, the distribution of the electrical conductivity, $\sigma _{E}$, and magnetic permeability, $\mu _{M}$ also evolves,together with the other hydrodynamic variables. Figure 7d–f show the final distributions of the Ni, Al and Fe respectively following complete solidification.

The various forces in the liquid metal flow act to homogenise the component distributions, as can be seen in Fig. 7. As the solidification front advances, the homogenisation caused by the mixing in the weld-pool is frozen into the domain. In this work, it is assumed that no diffusion occurs in the solid state, and at $t=1.67$ s the metallic substrate is once again fully solidified.

It is interesting to note that the amount of chemical homogenisation between the two alloys, as shown in Fig. 7, is not uniform. This is due to the highly transient nature of the advancing melt, and subsequent solidification fronts, meaning regions of the domain experience varied times in the liquid state. Although no experimental validation of the particular arc welding case is available, qualitatively the asymmetry in the fusion zone is in agreement with dissimilar welding cases reported in the literature as the alloy with the lower melting range experiences a greater degree of melting and dilution^45,46^.

## Discussion and conclusions

The availability of a multi-phase magneto-hydrodynamic framework allows the high-fidelity description of many interesting, and challenging physical phenomena at multiple length scales; from advanced manufacturing and fusion plasma’s, to astrophysical plasma and solar physics scenarios. In this work we present a multi-phase magneto-hydrodynamic formulation that can be used to simulate the behaviour of mixtures with large property gradients. An interface compression technique is used in a hybrid volume of fluid methodology that allows for the simulation of miscible and immiscible components using a one-fluid approach in the momentum equation. In the first instance the framework is validated against a single phase Hartmann flow analytical solution. It is shown that the framework faithfully matches the analytical solution for the Hartmann flow over a range of applied magnetic field strengths. The framework is further verified against experimentally measured Ar bubble rise trajectories subjected to various horizontally applied magnetic field strengths, as measured experimentally by Richter et al.^43^. The framework provides good agreement with the experimentally measured data both qualitatively and quantitatively; replicating the magnitude of the bubble oscillations and the distance between the directional inflection points of the bubbles. In the future, the proposed framework will be applied to more complex three dimensional validation cases for additional validation; however at the present time the authors believe the case of Richter et al. serves as an appropriate validation of the framework. Finally the framework is applied to the technologically relevant scenario of electrical arc welding of a dissimilar metal substrate. For the first time, a simulation of an electrical arc welding scenario is presented where no phenomenological fitting has occurred; the thermodynamic, and magneto-hydrodynamic evolution of the system is entirely determined by the boundary conditions of the respective fields, which are known quantities in advanced joining and deposition scenarios.

In this work the derived induction equation is fully coupled to the Navier–Stokes, phase, and energy transport equations. It is shown that through the projection method, the predicted magnetic $$\left( {\varvec{B}}\right)$$ field is maintained divergence free to machine accuracy. During the course of this study, two divergence cleaning approaches were investigated; the advection approach (where the non-zero magnetic divergence is advected out of the domain), and the projection method. The projection method was preferred by the authors, despite its slightly higher computational cost, as the solenoidal error magnitude could easily be maintained at negligible levels $$(\sim \times 10^{-12}$$ T m$$^{-1})$$ .

While the formulation may be used to simulate important advanced manufacturing processes such as electrical arc welding, plasma deposition processes, and to better understand the behaviour of fusion plasma interactions with liquid metal breeder/coolant blankets; for a more accurate prediction of these processes, the material properties, particularly the electrical conductivity, should be made a function of temperature in future work. The temperature dependence of the properties could be readily implemented, as the governing equations already account for position dependent properties through the various operators using the product rule. Similarly in the future, the solidus and liquidus temperatures, and thermal conductivity of any alloys investigated could be read from a coupled thermodynamic database, or lookup table; the current approach of linearly interpolating the pure-substance values to determine the mixture properties is known to work well for properties such as the density, but can over-predict the thermal conductivity.

Specific conclusions gleaned using the proposed framework are as follows:

- A robust mathematical description of multi-phase magneto-hydrodynamic flows is presented. Large property gradients, and the effect of these gradients on the system, are fully captured by the proposed framework. Melting and solidification state changes, as well as mass diffusion between the phases present is incorporated into the framework
- The framework is validated against well known analytical solutions, and experimentally measured multi-phase magneto-hydrodynamic flows. Excellent agreement is seen with comparison to the Hartmann flow analytical solution. The framework accurately predicts the multi-phase MHD flow of bubbles rising through a conducting liquid metal, for various applied field strengths, as measured by Richter et al.^43^.
- The framework is applicable to advanced manufacturing scenarios. The mixing behaviour in a dissimilar metal weld is observed, where Joule heating of the metallic substrate causes melting. Advanced joining and deposition processes may be understood without the requirement to fit a phenomenological heat source.

## Methods

In this section a magneto-thermal-hydrodynamic framework for describing multi-phase transport of heterogeneous mixtures with state transitions is outlined. Describing the evolution of a multi-phase magneto-thermal-hydrodynamic system requires equations that describe the fluid flow, energy transport, species transport, and finally magnetic field evolution in the entire domain. While the main focus of this work is the formulation of a multi-phase magnetic induction equation, that describes how the magnetic field evolves due to intrinsic velocity fields in multi-component substrates; for completeness the entire framework including the momentum and energy transport equations is shown below. The thermal and fluid dynamics will first be described, including the magnetic coupling terms, before a full derivation and implementation of the magneto-dynamics. The proposed framework fully describes multi-phase fluid flows and state change under the influence of magnetic fields.

### Thermal-fluid dynamics

The thermal-fluid dynamics of a multi-phase mixture is fully described by the fluid velocity, pressure, chemical species fraction and temperature fields through the conservation laws for linear momentum, composition and energy transport. This results in the following one-fluid formulation of the Navier–Stokes equation for the fluid velocity, for more details see^22,47,48^:

1 $$\begin{aligned} \frac{\partial \left( \rho {\varvec{U}}\right) }{\partial t}+\nabla \cdot \left( \rho {\varvec{U}}\otimes {\varvec{U}}\right) =-\nabla P+\nabla \cdot \varvec{\tau }+\left( {\varvec{J}}\times {\varvec{B}}\right) +\varvec{\varPhi }. \end{aligned}$$

where ${\varvec{U}}$ is the velocity, $\rho$ is the mass density, *P* is the fluid pressure, and $\varvec{\tau }$ is the viscous stress tensor and is given by,

2 $$\begin{aligned} \varvec{\tau }=\mu \left[ \nabla {\varvec{U}}+\left( \nabla {\varvec{U}}\right) ^{T}\right] -\frac{2}{3}\mu \left( \nabla \cdot {\varvec{U}}\right) {\varvec{I}}. \end{aligned}$$

In Eq. (1), the Lorentz force, ${\varvec{J}}\times {\varvec{B}}$, is included. The Lorentz force is a body force exerted on a fluid in the presence of a magnetic field, ${\varvec{B}}$, where ${\varvec{J}}$ is the electrical current density. Additional effects, such as buoyancy, surface tension and momentum damping due to solidification, are also included in $\varvec{\varPhi }$. Details are provided in Appendix 1.

An energy transport equation is used to solve for the temperature in the domain, and incorporates source terms resulting from the latent heat of fusion as well as Joule heating. Viscous dissipation and pressure transport are assumed negligible compared to the large convective, conductive and Ohmic heat fluxes typically encountered during MHD processes^12,49^. The energy transport equation is given by:

3 $$\begin{aligned} \frac{\partial \rho c_{p}T}{\partial t}+\nabla \cdot \left( {\varvec{U}}c_{p}\rho T\right) -\nabla \cdot \left( k\nabla T\right) =\frac{{\varvec{J}}\cdot {\varvec{J}}}{\sigma _{E}}+S_{h} \end{aligned}$$

where $c_{p}$, *k*, and $\sigma _{E}$ are the specific heat, thermal conductivity and electrical conductivity of the multi-phase mixture respectively. $S_{h}$ captures the latent heat effects due to melting and solidification of the substrate. In Eq. (3) ${\varvec{J}}\cdot {\varvec{J}}/{\sigma _{E}}$ is a source term that causes heating due to electrical conduction, known as Joule heating. This joule heating term similarly couples the evaluation of the electro-magnetic fields and the energy transport equation.

A system of advection–diffusion transport equations is utilised to describe the evolution of the multiple chemical mass fractions, $\alpha _{k}$, present in the mixture:

4 $$\begin{aligned} \frac{\partial \left( \rho _{k}\alpha _{k}\right) }{\partial t}+\nabla \cdot \left( \rho _{k}\alpha _{k}{\varvec{U}}\right) +\nabla \cdot \left( {\varvec{U}}_{c}\alpha _{k}\left( 1-\alpha _{k}\right) \right) =\nabla \cdot \left( \rho D_{k}\nabla \left( \frac{\rho _{k}\alpha _{k}}{\rho }\right) \right) \end{aligned}$$

The mixture effective diffusion coefficient $D_{k}$ is found from a weighted summation over the cross diffusion pairs, using the generalised Fickian model^50^. No geometric interface reconstruction or tracking is performed. A compressive velocity field, ${\varvec{U}}_{c}$, is instead superimposed in the vicinity of the interface to counteract numerical diffusion between the gaseous and metallic phases^51^. Additional details of the implementation can be found elsewhere^47^. The thermodynamic and transport properties of the heterogeneous mixture are computed using the mass phase fraction as a weighting factor, e.g. $\rho =\sum _{k}\rho _{k}\alpha _{k}$ for the mixture density. It is known that the treatment of interfaces with large property gradients can pose serious computational challenges, in the future the implementation of a harmonic interpolation approach for transport properties displaying the greatest spatial contrast will investigated^52^.

Note that total mass conservation is a consequence of Eq. (4), and is not solved additionally. For a detailed discussion of different blending and interpolation schemes and their applicability, please refer to^48,53^. Additional details of the thermal-fluid dynamics formulation of this framework are provided in Appendix 2.

### Magneto-hydro dynamics

The magneto-dynamics for a multi-phase fluid mixture, is completely described by Maxwell’s equations. In this section, we begin by stating the relevant Maxwell’s equations before applying simplifying assumptions and their rationale. The Ampere–Maxwell law, magnetic Gauss law, Faraday’s law and Ohm’s law for dynamic electro-magnetic fields are respectively given by:

5a $$\begin{aligned} \frac{\partial {\varvec{D}}}{\partial t}+{\varvec{J}}&=\nabla \times {\varvec{H}}, \end{aligned}$$

5b $$\begin{aligned} 0&=\nabla \cdot {\varvec{B}}, \end{aligned}$$

5c $$\begin{aligned} \frac{\partial {\varvec{B}}}{\partial t}&=-\nabla \times {\varvec{E}}, \end{aligned}$$

5d $$\begin{aligned} {\varvec{J}}&=\sigma _{E}\left[ {\varvec{E}}+{\varvec{U}}\times {\varvec{B}}\right] , \end{aligned}$$

where ${\varvec{D}}$ is the electric flux density, ${\varvec{E}}$ is the electric field intensity, ${\varvec{J}}$ is the electric current density, ${\varvec{U}}$ the fluid velocity, and $\sigma _{E}$ the electrical conductivity of the medium. Also required is the constitutive relation between the magnetic field, ${\varvec{B}}$, the magnetic field strength, ${\varvec{H}}$, and the magnetisation, ${\varvec{M}}$; ${\varvec{B}}=\mu _{M}{\varvec{H}}+{\varvec{M}}$, with $\mu _{M}$ the magnetic permeability of the medium, and $\mu _{0}$ the permeability of free space^54^. Materials considered in this work are free of electric polarisation, magnetisation and impressed currents, and thus have linear ${\varvec{B}}$ vs ${\varvec{H}}$ relations. Flows considered in this work also have a negligible velocity compared to the speed of light, $\left| {\varvec{U}}\right| \ll c$. Under these model assumptions, it can be shown that the Ampere–Maxwell law simplifies to $\nabla \times {\varvec{H}}={\varvec{J}}$, and the constitutive relation for the magnetic flux density simplifies to ${\varvec{B}}=\mu _{M}{\varvec{H}}$^55^.

The induction equation is derived from Eq. (5a-5d); usually in terms of ${\varvec{B}}$. First the curl of Eq. (5d) is taken, and then substituting Eqs. (5c) and (5a) resulting in the following expression:

6 $$\begin{aligned} \nabla \times \left( \frac{1}{\sigma _{E}}\nabla \times \frac{{\varvec{B}}}{\mu _{M}}\right) =-\frac{\partial {\varvec{B}}}{\partial t}+\nabla \times \left( {\varvec{U}}\times {\varvec{B}}\right) \end{aligned}$$

In previous work, on homogeneous systems, i.e. where $\mu _{M}$ and $\sigma _{E}$ are constant, Eq. (6) can be reduced to^8,11,12^:

7 $$\begin{aligned} \frac{\partial {\varvec{B}}}{\partial t}-\nabla \cdot \left( {\varvec{B}}\otimes {\varvec{U}}-{\varvec{U}}\otimes {\varvec{B}}\right) +\frac{1}{\mu _{M}\sigma _{E}}\nabla ^{2}{\varvec{B}}=0. \end{aligned}$$

However, in heterogeneous systems with strong contrast in $\mu _{M}$ and $\sigma _{E}$ across phase boundaries, a simplified form of the magnetic induction equation (Eq. 6) is not readily available due to a lack of identities that can transform the left hand side of Eq. (6) into a form that can be exploited by implicit solvers. Numerical solution of Eq. (6) in heterogeneous systems will result in severe time step restrictions, due to the left hand side of Eq. (6), which must be treated explicitly to guarantee a bounded solution. Certain identities can be used to expand these explicit terms with limited success as these will again lead to large magnitude $\nabla \mu _{M}$ and $\nabla \sigma _{E}$ terms in the resulting expressions that must again be treated explicitly.

As previously stated, the multi-phase nature of systems considered here means that material properties, such as $\sigma _{E}$ and $\mu _{M}$, display large spatial discontinuities at the boundaries between phases, such as the interface between a gaseous and metallic phases. For example, in the context of a dissimilar electrical arc welding process, the electrical conductivity, $\sigma _{E}$, of the metallic substrate is $\approx 10^{4}$ times that of the shielding plasma. Similarly the magnetic permeability, $\mu _{M}$, of the metallic substrate is $\approx 10^{2}$ times that of the shielding plasma.

In this work, the magnetic induction equation is instead formulated in terms of the magnetic field strength, ${\varvec{H}}$. The resulting field equation is given by:

8 $$\begin{aligned} \frac{\partial \mu _{M}{\varvec{H}}}{\partial t}-\nabla \cdot \left[ \mu _{M}\left( {\varvec{H}}\otimes {\varvec{U}}-{\varvec{U}}\otimes {\varvec{H}}\right) \right] +\nabla \cdot \left[ \frac{1}{\sigma _{E}}\left( \nabla {\varvec{H}}^{\mathrm {T}}-\nabla {\varvec{H}}\right) \right] =0. \end{aligned}$$

Details of the derivation are provided in Appendix 3. The formulation of the induction equation in terms of ${\varvec{H}}$ permits more of the resulting terms in the expression to be treated implicitly, and therefore the overall numerical implementation is more robust, with less stringent time-step limitations. The diffusive term in Eq. (8) is treated implicitly, while the term containing the gradient transpose is treated explicitly^56^ .

The coupling terms between the induction equation and the momentum and energy transport equations, namely the Lorentz force and Joule heating terms, are implemented explicitly in this work. The Lorentz force term as ${\varvec{J}}\times {\varvec{B}}=\left( \nabla \times {\varvec{H}}\right) \times \mu _{M}{\varvec{H}}$, and the Joule heating term as ${\left( \nabla \times {\varvec{H}}\right) \cdot \left( \nabla \times {\varvec{H}}\right) }/{\sigma _{E}}$.

### Numerical implementation

The governing PDE’s for the magnetic field strength (Eq. 8), fluid velocity (Eq. 1), temperature (Eq. 3) and mixture fractions (Eq. 4), are discretized within the OpenFOAM library using a cell centred finite volume approach^22^. The discretised PDE’s are solved semi-implicitly at every time step. First the velocity and pressure fields are solved iteratively until convergence of the incompressibility constraint is reached using a PISO (pressure implicit with splitting of operators) approach with the temperature and composition fields updated within each PISO iteration^22^. The pressure-Poisson equation, constructed to correct the velocity field in the PISO approach is given by,

9 $$\begin{aligned} \nabla \cdot \left( A_{D}^{-1}\nabla P\right) =\nabla \cdot \varvec{U^{*}} \end{aligned}$$

where ${1}/{A_{D}}$ are the diagonal entries of the momentum matrix equation. This is followed by the solution of the modified magnetic induction equation (Eq. 8) for the field strength, ${\varvec{H}}$. While the magnetic solenoidal constraint, $\nabla \cdot {\varvec{B}}=0$, is exactly satisfied at all times with a consistent initial condition in ${\varvec{H}}$. Numerical time stepping methods can introduce non-solenoidal perturbations to the solution. A non-solonoidal component in ${\varvec{B}}$ can introduce an erroneous force, parallel to the magnetic field in the momentum equation, causing nonphysical effects in the flow^57^. In the multi-phase cases presented here this would be particularly damaging to the solution around the regions where gradients in $\mu _{M}$ exist. In order to eliminate any spurious non-solenoidal component of the magnetic field, introduced by numerical time stepping, a scalar Lagrange multiplier field, $P_{H}$, analogous to the fluid pressure, *P*, is introduced. An approach, very similar to the approach used by Rhie and Chow for the velocity and pressure field coupling^58^, is then used to correct the magnetic field strength solution at the end of every time step.

Given a magnetic field solution, $\varvec{B^{*}}=\mu _{M}\varvec{H^{*}}$, containing a spurious non-solonoidal component, can be decomposed unambiguously into the sum of a curl and a gradient

10 $$\begin{aligned} \varvec{B^{*}}=\nabla \times {\varvec{A}}+\nabla P_{H} \end{aligned}$$

where the curl of the vector potential ${\varvec{A}}$ contains the physically meaningful, solonoidal part of $\varvec{B^{*}}$. Taking the divergence of both sides results in a Poisson equation, $\Delta P_{H}=\nabla \cdot \varvec{B^{*}}$, that can be readily solved for the scalar field $P_{H}$. The numerical divergence of $\varvec{B^{*}}$ will be exactly zero if the Laplace operator in $\Delta P_{H}$ is evaluated in two steps as a divergence of a gradient^21^. It is then easy to correct the magnetic field by the gradient of this scalar field, ${\varvec{B}}=\varvec{B^{*}}-\nabla P_{H}$, to ensure a divergence free magnetic field^21^. As found by previous authors^21^, the choice of the divergence cleaning approach is inconsequential, as long as the solonoidal constraint is maintained. Alternative approaches to maintain the divergence free ${\varvec{B}}$ field include advecting the non-zero ${\varvec{B}}$ divergence contributions out of the boundaries of the computational domain. Recalling that ${\varvec{H}}={\varvec{B}}/\mu _{M}$, is used as the primary solution field in the present formulation, the projection method is modified accordingly:

11 $$\begin{aligned} \nabla \cdot \left( A_{DH}^{-1}\nabla P_{H}\right)&=\mu _{M}\nabla \cdot \varvec{H^{*}}+\nabla \mu _{M}\cdot \varvec{H^{*}},\quad \text {and} \nonumber \\ {\varvec{H}}&=\varvec{H^{*}}-\frac{1}{\mu _{M}}\nabla P_{H} \end{aligned}$$

The projection method utilised in this work is generally preferred, as the error produced in the solenoidal constraint is significantly reduced over advection approaches^59^. $P_{H}$ holds the divergence error which tends to zero as the system of equations are iterated to convergence. However, it need not actually reach zero, as it will hold a form of discretisation error representing the difference between the ${\varvec{H}}$ field fluxes and the face-interpolate of the cell-centre ${\varvec{H}}$ field. Tests have shown this error to be small and therefore $P_{H}$ to be small.

The mixture transport equations for $\varvec{\alpha }=\left[ \alpha _{1},\alpha _{2},\ldots ,\alpha _{N}\right]$ are handled explicitly; requiring extremely small Courant numbers. It should be noted that for a single component flow, i.e. one with no variation in $\mu _{M}$, the divergence of the ${\varvec{B}}$ field would simply be equal to the divergence of the ${\varvec{H}}$ field (multiplied by the magnetic permeability), as $\nabla \mu _{M}$ would be zero in this case. The multi-dimensionsal limiter for explicit solution (MULES) approach is utilised for the solution of the multi-phase mixture transport equation. The ${\varvec{J}}\times {\varvec{B}}$ term in (1) is handled explicitly in the momentum equation. The overall solution procedure used is shown in Algorithm 1.

A semi-implicit approach is used to solve for the momentum and magnetic field evolution as the magnetic field in the cases presented evolves at a much slower rate than the momentum field. A second order accurate backward Euler scheme is used for the time integration. For the spatial discretisation a second order least-squares scheme is utilised. The numerical errors in $\nabla \cdot {\varvec{U}}$ and $\nabla \cdot \left( \mu _{M}{\varvec{H}}\right)$ generally arise as local errors of opposite sign which are dealt with through conjugate gradient solvers efficiently. Therefore for the Poisson pressure and magnetic pressure problems, these conjugate gradient are utilised in this work. For the energy transport equation, a smooth solver is utilised.

## Appendix 1: Particulars of the momentum equation

The term $\varvec{\Phi }$ in the momentum equation gathers the buoyancy, $\varvec{F_{g}}$, surface forces , $\varvec{F_{s}}$, and the Darcy source term used to dampen the velocity field upon solidification, $\varvec{S_{m}}$, $\varvec{\varPhi }=\varvec{F_{s}}+\varvec{F_{g}}+\varvec{S_{m}}$. The forms of the expressions used for the buoyancy and Darcy source terms in this work are given as,

12a $$\begin{aligned} \varvec{F_{g}}&=\rho g\beta \left( T-T_{m}\right) \end{aligned}$$

12b $$\begin{aligned} \varvec{S_{m}}&=K_{1}\frac{\left( 1-\epsilon _{1}\right) ^{2}}{\epsilon _{1}^{3}+K_{2}}{\varvec{U}} \end{aligned}$$

where *g* is the gravitational constant, $\beta$ is the thermal expansion coefficient of the mixture, and $T_{m}$ is the mixture liquidus temperature. $\epsilon _{1}$is the fluid fraction where a value of 1 corresponds to fully melted, and a value of 0 corresponds to completely solidified. In the Darcy source term, the constants $K_{1}$ and $K_{2}$ are chosen as $10^{9}$ and $10^{-12}$ respectively to dampen ${\varvec{U}}$ to *numerically* zero in the solidified regions of the domain^60–62^. For the surface forces, the expression for $\varvec{F_{s}}$ is given as,

13 $$\begin{aligned} \varvec{F_{s}}=-\sum _{k=0}^{N}\sum _{l\ne k}^{N}\left[ \sigma _{kl}\nabla \cdot \left( \frac{\varvec{\varvec{\psi }}}{\left| \varvec{\varvec{\psi }}\right| }\right) \left( \frac{\varvec{\varvec{\psi }}}{\left| \varvec{\varvec{\psi }}\right| }\right) +\frac{d\sigma _{kl}}{dT}\left( \nabla T-\frac{\varvec{\varvec{\psi }}}{\left| \varvec{\varvec{\psi }}\right| }\left( \frac{\varvec{\varvec{\psi }}}{\left| \varvec{\varvec{\psi }}\right| }\cdot \nabla T\right) \right) \right] \left| \varvec{\varvec{\psi }}\right| \end{aligned}$$

where $\varvec{\varvec{\psi }}=\alpha _{l}\nabla \alpha _{k}-\alpha _{k}\nabla \alpha _{l}$. The surface force term is comprised of two contributions; the normal surface tension contribution, and a contribution arising from the temperature dependence of the surface tension. The surface tension is implemented using the continuum surface force model^48, 63^. The temperature dependence of the surface tension, also known as the Marangoni effect, causes the generation of shear flow at the interface of liquids under a temperature gradient, where the flow direction is from the higher temperature region towards the lower temperature region. The implementation used in this work is validated against a well known analytical solution^64^.

## Appendix 2: Particulars of the energy transport equation

The latent heat source term for the multi-phase mixture is,

14 $$\begin{aligned} S_{h}=-L\left( \frac{\partial \rho \epsilon _{1}}{\partial t}+\nabla \cdot \left( {\varvec{U}}\rho \epsilon _{1}\right) \right) \end{aligned}$$

where *L* is the latent heat of fusion of the mixture. The liquid fraction of the metallic phases, $\epsilon _{1}$, is determined by the local temperature of the mixture relative to the mixture solidus and liquidus temperatures:

15 $$\begin{aligned} \epsilon _{1}=\left\{ \begin{array}{l} 0\,\,\,\,T<T_{s}\\ {(T-T_{s})}/{(T_{l}-T_{s})}\,\,\,\,T_{s}<T<T_{l}\\ 1\,\,T>T_{l} \end{array}\right. \end{aligned}$$

where $T_{s}$ and $T_{l}$ are the sums of the solidus and liquidus temperatures of the pure materials, weighted by the phase fractions of the components present in a given region. The solidus and liquidus temperatures of the pure substances is assumed to be $\pm 5\,K$ respectively.

## Appendix 3: Derivation of the modified magnetic induction equation

Taking the curl of Ohm’s law (Eq. 5d) yields the following*:*

$$\begin{aligned} \nabla \times \left[ \frac{{\varvec{J}}}{\sigma _{E}}\right] =\nabla \times \left[ {\varvec{E}}+{\varvec{U}}\times {\varvec{B}}\right] \end{aligned}$$

Substituting in Faradays law, which relates the time derivative of the magnetic flux density to the curl of the electric field, the following expression is obtained:

$$\begin{aligned} \nabla \times \left[ \frac{{\varvec{J}}}{\sigma _{E}}\right] =\frac{-\partial {\varvec{B}}}{\partial t}+\nabla \times \left[ {\varvec{U}}\times {\varvec{B}}\right] \end{aligned}$$

Substituting in the Ampere–Maxwell law for the current density (with the assumption that $\left| {\varvec{U}}\right| \ll c$) and recalling that ${\varvec{B}}=\mu _{M}{\varvec{H}}$, from the constitutive relation between the magnetic flux density ${\varvec{B}}$ and field strength ${\varvec{H}}$:

$$\begin{aligned} \nabla \times \left[ \frac{\nabla \times {\varvec{H}}}{\sigma _{E}}\right] =\frac{-\partial \mu {\varvec{H}}}{\partial t}+\nabla \times \left[ {\varvec{U}}\times \mu {\varvec{H}}\right] \end{aligned}$$

$$\begin{aligned} \frac{\partial \mu {\varvec{H}}}{\partial t}-\nabla \times \left[ {\varvec{U}}\times \mu {\varvec{H}}\right] =-\nabla \times \left[ \frac{\nabla \times {\varvec{H}}}{\sigma _{E}}\right] \end{aligned}$$

Finally using the vector identity $\nabla \times \left( {\varvec{A}}\times {\varvec{B}}\right) =\nabla \cdot \left( \varvec{BA^{T}}-\varvec{AB^{T}}\right)$, and knowing that $\varvec{BA^{T}}=\varvec{AB}$, the induction equation can be expressed as:

$$\begin{aligned} \frac{\partial \mu {\varvec{H}}}{\partial t}-\nabla \cdot \left[ \mu _{M}\left( {\varvec{H}}\otimes {\varvec{U}}-{\varvec{U}}\otimes {\varvec{H}}\right) \right] =-\nabla \cdot \left[ \frac{1}{\sigma _{E}}\left( \nabla {\varvec{H}}^{\mathrm {T}}-\nabla {\varvec{H}}\right) \right] \end{aligned}$$
